# Evaluation of Clinicopathological and Risk Factors for Nonmalignant *H. Pylori* Associated Gastroduodenal Disorders in Iraqi Patients

**DOI:** 10.3889/oamjms.2015.113

**Published:** 2015-11-05

**Authors:** Ali Ibrahim Ali AL-Ezzy

**Affiliations:** *Department of Pathology, College of Veterinary Medicine, Diyala University, Baquba, Diyala Province, Iraq*

**Keywords:** Risk factors, *H. pylori*, Cag A, gastroduodenal disorders, clinicopathological parameters

## Abstract

**AIM:**

To determine the risk factors associated with *H. pylori* infection and possible correlation with clinicopathological parameters.

**MATERIAL AND METHODS:**

Gastroduodenal biopsies were examined by rapid urease test and Gram staining. Cag A cytotoxin was detected by *in situ* hybridization.

**RESULTS:**

Risk of *H. pylori* acquisition reported as following: Males have 1.38 fold, rural residents have 0.63 fold, Nonsmokers have 0.39 fold, mild smokers have 18 fold, and moderate smokers have 1.4 fold while heavy smokers have 1 fold. A person who’s in contact with animals has 1.52 fold risks. Illiterates and patients with primary education have 5.36 & 3 fold risk respectively. Patients under proton pump inhibitor (PPI) therapy have 1.02 fold. Patients under NSAID therapy have 3.48 fold while nonalcoholic Patients have 0.75 fold. Patients using tap water have 0.45 fold risk. *H. pylori* infection positively correlated with age, weight loss, and heartburn. *H. pylori* inversely correlated with endoscopic diagnosis, Cag A positivity, and education level. Cag A positivity correlated with animal contact and NSAID usage.

**CONCLUSIONS:**

Several life style factors, education, animal contact, using of PPI, and NSAIDs increase the risk of *H. pylori* infection. Weight loss and heartburn cardinal signs for *H. pylori* infection. Endoscopic diagnosis and clinicopathological parameters not strictly associated with Cag A positivity.

## Introduction

Helicobacter pylori infection is conventional chronic bacterial infection around the world [1]. The pathogen is the most common cause of morbidity and mortality in upper digestive tract diseases and has a strong association with a variety of gastric pathologies, including type B antral gastritis, peptic ulcer, gastric MALT lymphoma, and gastric adenocarcinoma [2, 3].

Extra-alimentary ailments such as coronary disease, myocardial infarction, migraine, dermatological disorders, iron deficiency anemia, and some autoimmune diseases also developed due to *H. pylori* [4]. It has been shown that 50% adult in developed countries and 90% adults in developing countries were positive of serum antibodies against *H. pylori* [4]. The critical period at which *H. pylori* is acquired, is during the childhood, especially in the developing countries and areas of overcrowding and socioeconomic deprivation [5].

*H. pylori* have developed a repertoire of functions for survival in the harsh gastric niche, including acid tolerance, motility, adherence, immune evasion and mechanisms for adaptive evolution. These features are all involved in the interplay between the host and the bacterium and may influence acquisition and persistence of infection. Bacterial acid tolerance and motility play critical role in gastric colonization [6]. The Objective of this study was to determine the risk factors associated with *H. pylori* infection and possible correlations between Clinicopathological parameters with these risk factors.

## Material and Methods

### Patients

In this cross sectional, hospital based study, 106 patients, age range 15-80 years, mean ± SD (44.70 ± 18.260) with clinical indications for gastroduodenoscopy during February 2013 to June 2014 were studied. This study conducted according to the principles of Helsinki declaration. Dully-filled consent form obtained from all patients before endoscopy in Gastroenterology Department of Baqubah Teaching Hospital in Diyala Province - Iraq. Approval of ethical review Committee of College of Medicine, Diyala University, Iraq; taken prior to initiation of the work. Patients were excluded in the following circumstances: having a history of previous gastric surgery, recent or active gastrointestinal bleeding, patients with treated with antibiotics or colloidal bismuth compounds for past one month, if the informed consent was not obtained [7].

### Methods

After topical pharyngeal anesthesia for overnight fasted Patients, A sterile flexible endoscope introduced for full investigation of Stomach and duodenum. Six endoscopic biopsies obtained from congested, inflamed or erosive lesions via sterile biopsy forceps. The samples for Gram staining procedure were retired from the biopsy forceps using a sterile needle, and placed in an Eppendorf tube containing 0.5 mL of sterile saline as a means of transportation [7]. Samples for rapid urease test placed in separate vial, previously identified, containing the appropriate medium for test.

The RUT performed. This test was performed with a homemade solution with 1 ml distilled water, one drop of 1% phenol red (pH = 6.5), and 100 mg urea, prepared just before endoscopy. One antral sample placed in the solution and tube then incubated at 37°C. The test considered positive when the color changed from yellow, pink to red within 24 hours [8]. Biopsy sample placed in sterile glass slide with a drop of normal saline and teased with sterile scalpel to make smaller fragments of tissue then another sterile glass slide was placed over the teased first tissue and the tissue was crushed between the two glasses then stain by Gram’s staining. Existence of Gram negative spiral bacteria embedded in the tissue cells was diagnostic for *H. Pylori* [9]. true positive results were considered if a combination of urease test and Gram stain give positive results for a single biopsy specimen [10]. *H pylori* cytotoxin-associated gene A (*CagA*) was determined using biotinylated DNA probe (*H. pylori* / *CagA* Gene, Cat. No.: IH-60061, (Maximbiotech product Catalog # SP-10216) as previously described [7]. The dependent Scoring system for smoking habits (non-smoker = 0 cigarette per day; mild smoker = 10 cigarette per day; moderate smoker = 11-20 cigarette per day; heavy smoker = >20 cigarette per day) [11]. Scoring system for patients education (Illiteracy; primary; secondary; university) [11]. The dependent Scoring system for Epigastric pain (negative, mild, moderate) and for heart burn (negative, mild, moderate) according to [12] with modification.

### Statistical analysis

Data analysis performed using the following tests: Frequency of variables express as percentage. Pepsinogen I, II and gastrin values express as mean ± standard deviation, (Mean ± SD). Chi square and Pearson test for correlation used for non-categorical data. Estimated risk (Odd ratio) and the corresponding 95% confidence intervals for all tests were calculated using MedCalc statistical software, Version 13.1.1, Belgium. The level of significance was 0.05 (two-tail) in all statistical testing; significant of correlations include also 0.01 (two-tail). Statistical analysis was performed using also SPSS for windows TM version 17.0, and Microsoft EXCEL for windows 2010.

## Results

Demographic features of 106 patients are shown in Table 1.

**Table 1:** Demographic Risk Factors for *H. pylori* infection

Age (years)		*H. pylori* test					Estimated risk		X2P value	Correlation among *H. pylori* and risk factors		Correlation among *H. pylori* Cag A cytotoxin and risk factors	

		*H. pylori* Positive			*H. pylori* Negative	Total No. (%)	Odd ratio	95% Confidence Interval	
	
		Total positive	Cag A cytotoxin positive	Cag A cytotoxin negative						r	P value	r	P value
15-23		3 (2.83%)	2 (1.88%)	1 (0.94%)	5 (4.72%)	8 (7.55%)	0.64	0.0101- 40.0759	0.037***	0.229*	0.018	0.131

24-32		16 (15.09%)	8 (7.55%)	8 (7.55%)	7 (6.60%)	23 (21.70%)	2.2	0.0397 - 121.7979				

33-41		24 (22.64%)	17 (16.04%)	7 (6.60%)	6 (5.66%)	30 (28.30%)	3.77	0.0681 - 208.7469				

42-50		8 (7.55%)	5 (4.72%)	3 (2.83%)	2 (1.89%)	10 (9.43%)	3.4	0.0528- 219.1394				

51-59		7 (6.60%)	2 (1.88%)	5 (4.72%)	3 (2.83%)	10 (9.43%)	2.14	0.0348 -131.9417					0.181

60-68		4 (3.77%)	2 (1.88%)	2 (1.88%)	1 (0.94%)	5 (4.72%)	3	0.0394 -228.6827				

69-77		15 (14.15%)	9 (8.49%)	6 (5.66%)	2 (1.89%)	17 (16.04%)	6.20	0.0986- 389.9085				
78-86		3 (2.83%)	3 (2.83%)	0 (0%)	0 (0%)	3 (2.83%)	7	0.0514 - 953.2624				

Total		80 (75.47%)	48 (45.3%)	32 (30.19%)	26 (24.53%)	106 (100%)						

Gender	Male	43 (40.57%)	29 (27.4%)	14 (13.21%)	16 (15.09%)	59 (55.7%)	1.38	0.557-3.40	0.24	0.067	0.492	-0.087	0.375

	Female	37 (34.91%)	19 (17.92%)	18 (16.98%)	10 (9.43%)	47 (44.3%)							

Residence	Rural	46 (43.39%)	28	18 (16.98%)	12 (11.32%)	58 (54.7%)	0.63	0.260-1.54	0.33	-0.098	0.317	-0.066	501

	Urban	34 (32.08%)	20 (18.87%)	14 (13.21%)	14 (13.21%)	48 (45.3%)							

* Correlation is significant at the 0.05 level (2-tailed); ** Correlation is significant at the 0.01 level (2-tailed);

*** ANOVA.

Significant difference was detected among age groups in *H. pylori* infection (p < 0.001). Significant positive correlation was reported between age groups and *H. pylori* infection (r = 0.229, p value = 0.018). While no significant correlation was reported between age groups and infection with Cag A cytotoxin producing *H. pylori* (r = 0.131, p value = 0.181) as shown in Table 1. Males represent 55.7% vs. females 44.3% (Table 1). Males have 1.38 fold risk of *H. pylori* acquisition compared with females without significant difference between males and females infected with *H. pylori* (p > 0.05). There was no significant correlation between *H. pylori* infection and gender of patient (r = 0.067, p value = 0.492). No significant correlation was reported between gender and infection with Cag A cytotoxin producing *H. pylori* (r= -0.087, p value = 0.375).

Residence in rural was 54.7% vs. 45.3% in urban areas. Peoples residence in rural areas have 0.63 fold risk of *H. pylori* acquisition compared with those from urban without significant difference between patients from rural and urban areas infected with *H. pylori* (p > 0.05). There was no significant correlation between *H. pylori* infection and residence of patient (r = -0.098, p value = 0.317) (Table 1). No significant correlation was reported between residence and infection with *H. pylori* Cag A positive (r = -0.066, p value = 501) as shown in Table 1.

Education and behavioral risk factors for *H. pylori* infection are shown in Table 2. Regarding education level 27.36% of *H. pylori*, positive cases were illiterate, 15.09% primary education and university education; 17.92% finished secondary education. Illiterates and patients with primary education have 5.36 & 3 fold risk for acquisition of *H. pylori* infection. While those at secondary and university education level have, 0.42 and 1.24 fold risk respectively without significant differences (p > 0.05). Inverse correlation between *H. pylori* infection and education levels of patients was detected (r = -0.225, p value = 0.020). Inverse significant correlation between *H. pylori* Cag A positive and education levels (r = -0.331, p value = 0.001) as shown in Table 2.

**Table 2:** Education and Behavioral Risk Factors for *H. pylori* infection

Parameters			*H. pylori* test					Estimated risk		X2 P value	Correlation among *H. pylori* and risk factors		Correlation among *H. pylori* Cag A cytotoxin and risk factors	

			*H. pylori* Positive			*H. pylori* Negative	Total No. (%)	Odd ratio	95% Confidence Interval	
	
			Total positive	Cag A cytotoxin positive	Cag A cytotoxin negative						r	P value	r	P value
Education level		Illiteracy	29 (27.36%)	20 (18.87%)	9 (8.49%)	5 (4.72%)	34 (32.1%)	5.36	0.0959 - 300.0519	0.16	-0.225*	0.020	-0.331	0.001

		Primary	16 (15.09%)	13 (12.26%)	3 (2.83%)	2 (1.89%)	18 (17.0%)	3	0.150-59.890					

		Secondary	19 (17.92%)	10 (9.43%)	9 (8.49%)	10 (9.43%)	29 (27.4%)	0.42	0.040-4.334					

		University	16 (15.09%)	5 (4.72%)	11 (10.38%)	9 (8.49%)	25 (23.6%)	1.24	0.166-9.253										

Smoking		Nonsmoker	50 (47.17%)	31 (29.2%)	19 (17.92%)	17 (16.04%)	67 (63.2%)	0.39	0.112-1.73	0.000	-0.030	0.764	-0.077	0.436

		Mild smoker	22 (20.75%)	14 (13.21%)	8 (7.55%)	5 (4.72%)	27 (25.5%)	18	1.562-207					

		Moderate smoker	7 (6.60%)	2 (1.88%)	5 (4.72%)	3 (2.83%)	10 (9.4%)	1.4	0.876-2.237					

		Heavy smoker	1 (0.94%)	1 (0.94%)	0 (0%)	1 (0.94%)	2 (1.9%)	1	0.0108 - 92.4276					

Animal contact		Positive	25 (23.58%)	22 (20.75%)	3 (2.83%)	6 (5.66%)	31 (29.2%)	1.52	0.542-4.23	0.000	0.077	0.431	0.332	0.001

		Negative	55 (51.89%)	26 (24.53%)	29 (27.4%)	20 (18.87%)	75 (70.8%)							

Drugs	PPI	Negative	71 (66.98%)	40 (37.74%)	31 (29.2%)	23 (21.70%)	94 (88.7%)	1.02	0.242-3.897	0.000	-0.004	0.968	0.153	0.116

		Positive	9 (8.49%)	8 (7.55%)	1 (0.94%)	3 (2.83%)	12 (11.3%)							

	NSAID	Negative	62 (58.49%)	34 (32.1%)	28 (26.42%)	24 (22.64%)	86 (81.1%)	3.48	0.751-16.169	0.000	0.163	0.095	0.239	0.01

		Positive	18 (16.98%)	14 (13.21%)	4 (3.77%)	2 (1.89%)	20 (18.9%)							

Alcohol		Negative	79 (74.52%)	57 (53.77%)	22 (20.75%)	26 (24.53%)	105 (99.1%)	0.75	0.674-0.840	0.000	0.056	0.056	-0.089	0.365

		Positive	1 (0.94%)	1 (0.94%)	0 (0%)	0 (0%)	1 (0.9%)							

Water source		Spigot water	74 (69.81%)	43 (40.57%)	31 (29.2%)	22 (20.75%)	96 (90.6%)	0.45	0.115-1.72	0.000	-0.116	0.236	0.031	0.756

		Filtrated water	6 (5.66%)	5 (4.72%)	1 (0.94%)	4 (3.77%)	10 (9.4%)							

* Correlation is significant at the 0.05 level (2-tailed); ** Correlation is significant at the 0.01 level (2-tailed).

Nonsmokers represent 63.2%, 25.5% mild, 9.4% moderate, 1.9% heavy smokers, respectively. Nonsmoker have 0.39 fold risk for *H. pylori* acquisition, mild smokers have 18 fold risk, moderate smokers have 1.4 fold risk while heavy smokers have 1 fold risk of *H. pylori* acquisition. There was significant difference between smoker patients infected with *H. pylori* (p < 0.05). No significant correlation between *H. pylori* infection and smoking (r = -0.030, p value = 0.764) (Table 2). No significant correlation was reported between smoking and infection with *H. pylori* Cag A positive (r = -0.077, p value = 0.436).

Patients usually in contact with animals represent 29.2% among them 23.58% proved to have *H. pylori* infection, while 51.89% of patients with positive *H. pylori* have no contacts with animals. Peoples who are in contact with animals have 1.52 fold risk of *H. pylori* acquisition compared with those who do not in direct contact with animals with significant differences (p < 0.05). No significant correlation between *H. pylori* infection and contact with animals (r=0.077, p value=0.431) was found (Table 2). Significant correlation was reported between contact with animals and infection with *H. pylori* Cag A positive (r = 0.332, p value = 0.001).

As shown in Table 2, a total of 66.98% of patients with positive *H. pylori* never administrates proton pump inhibitor (PPI) and 8.49% of patients with history of PPI administration. Patients under PPI therapy have 1.02 fold to get *H. pylori* infection compared with those do not use PPI with significant difference (p < 0.05). No correlation between PPI administration and *H. pylori* infection (r = -0.004, p value = 0.968) (Table 1). No correlation was reported between PPI administration and infection with *H. pylori* Cag A positive (r = 0.153, p value = 0.116).

A total of 62 (58.49%) of patients with positive *H. pylori* never administrates NSAID and 24 (22.64%) of patients with history of NSAID administration. Patients under NSAID therapy have 3.48 fold to get *H. pylori* infection compared with those dot use NSAID with significant difference (p < 0.05). No significant correlation between NSAID administration and *H. pylori* infection (r = -0.163, p value = 0.095). Signi-ficant correlation reported between NSAID admini-stration and infection with *H. pylori* Cag A positive (r = 0.239, p value = 0.01), as shown in Table 2.

A total of 74.52% of patients with positive *H. pylori* never administrates alcohol and 24.53% with history of alcohol intake. Non alcoholics have 0.75 fold to get *H. pylori* infection compared with alcoholics with significant difference (p < 0.05). No significant correlation between alcohol intake and *H. pylori* infection (r = 0.056, p value = 0.056). No significant correlation was reported between alcohol intake and infection with *H. pylori* Cag A positive (r = -0.089, p value = 0.365) as shown in Table 2.

A total of 69.81% of patients with positive *H. pylori* administrates spigot water and 20.75% with spigot water administration give negative results. Patients administrates spigot water have 0.45 fold to get *H. pylori* infection compared with filtrated water with significant difference (p < 0.05). No significant correlation between spigot water administration and *H. pylori* infection (r = -0.116, p value = 0.236) as shown in Table 2. No significant correlation was reported between spigot water administration and infection with *H. pylori* Cag A positive(r = 0.031, p value = 0.756).

As shown in Table 3, Gastritis was detected in 37.7%, gastropathy 27.36%, gastric ulcer 16.98%, duodenal ulcer 12.26%, and Duodenitis 5.7% of total patients (Table 3). As shown in Table 3, *H. pylori* infection was detected in 22.64% in 33-41 years, the main clinical presentation was gastropathy 10.37% and gastritis 7.55% and they have 3.77 fold risks to get infection than those with *H. pylori* negative gastropathy or gastritis. Cag A cytotoxin was detected insitu in 16.04% of *H. pylori* positive cases. The second age group in *H. pylori* exposure was 24-32 years, 15.09%, presented with gastritis 4.72% and gastropathy 3.77% (Table 2). They have 2.2 fold risks to get infection than those with *H. pylori* negative gastropathy or gastritis in the same age group. Cag A cytotoxin detected in 7.55% of *H. pylori* positive cases.

**Table 3:** Distribution of Endoscopic diagnosis according to patient’s age

Age (Years)	Endoscopic diagnosis												

	Gastric ulcer		Gastritis		Gastropathy		Duodenal ulcer		Duodenitis		Total H.pylori		Total no. of patients

	*H. pylori* +ve	*H. pylori* -ve	*H. pylori* +ve	*H. pylori* -ve	*H. pylori* + ve	*H. pylori* -ve	*H. pylori* +ve	*H. pylori* -ve	*H. pylori* +ve	*H. pylori* -ve	Positive	Negative
15-23	0 (0%)	0 (0%)	1 (0.94%)	2 (1.89%)	2 (1.89%)	2 (1.89%)	0 (0%)	1 (0.94%)	0 (0%)	0 (0%)	3 (2.83%)	5 (4.72%)	8 (7.55%)

24-32	3 (2.83%)	0 (0%)	5 (4.72%)	3 (2.83%)	1 (0.94%)	4 (3.77%)	4 (3.77%)	0 (0%)	3 (2.83%)	0 (0%)	16 (15.09%)	7 (6.60%)	23 (21.70%)

33-41	1 (0.94%)	1 (0.94%)	8 (7.55%)	3 (2.83%)	11 (10.37%)	2 (1.89%)	3 (2.83%)	0 (0%)	1 (0.94%)	0 (0%)	24 (22.64%)	6 (5.66%)	30 (28.30%)

42-50	2 (1.88%)	0 (0%)	3 (2.83%)	1 (0.94%)	4 (3.77%)	0 (0%)	0 (0%)	0 (0%)	0 (0%)	0 (0%)	8 (7.55%)	2 (1.89%)	10 (9.43%)

51-59	1 (0.94%)	1 (0.94%)	5 (5.66%)	1 (0.94%)	0 (0%)	2 (1.89%)	0 (0%)	0 (0%)	0 (0%)	0 (0%)	7 (6.60%)	3 (2.83%)	10 (9.43%)

60-68	2 (1.88%)	0 (0%)	0 (0%)	1 (0.94%)	1 (0.94%)	0 (0%)	0 (0%)	0 (0%)	1 (0.94%)	0 (0%)	4 (3.77%)	1 (0.94%)	5 (4.72%)

69-77	7 (6.60%)	0 (0%)	5 (4.72%)	2 (1.89%)	0 (0%)	0 (0%)	2 (1.89%)	0 (0%)	1 (0.94%)	0 (0%)	15 (14.15%)	2 (1.89%)	17 (16.04%)

78-86	0 (0%)	0 (0%)	0 (0%)	0 (0%)	0 (0%)	0 (0%)	3 (2.83%)	0 (0%)	0 (0%)	0 (0%)	3 (2.83%)	0 (0%)	3 (2.83%)

Total	16 (15.09%)	2(1.89%)	27 (25.47%)	13 (12.26%)	19 (17.92%)	10 (9.43%)	12 (12.26%)	1 (0.94%)	6 (5.66%)	0 (0%)	80 (75.47%)	26 (24.53%)	106 (100%)

	18 (16.98%)		40 (37.74%)		29 (27.36%)		13 (12.26%)		6 (5.66%)		106 (100%)		

The third more exposed age group was 69-77 years in which *H. pylori* detected in 14.15% mostly with gastric ulcer 6.60% and gastritis 4.72%. They have 6.20 fold risks to get infection than those with *H. pylori* negative gastric ulcer or gastritis in the same age group. Cag A cytotoxin was detected in 8.49% of *H. pylori* positive cases. *H. pylori* Infection detected among 42-50 years in 7.55%, mostly with gastropathy 3.77% and gastritis 2.83%. They have 3.4 fold risks to get infection than those with *H. pylori* negative gastropathy or gastritis in the same age group. Cag A cytotoxin detected in 4.72% of *H. pylori* positive cases. *H. pylori* Infection detected among 51-59 years in 6.60%, mostly with gastritis 5.66%. They have 2.14 fold risk to get infection than those with *H. pylori* negative gastritis in the same age group. Cag A cytotoxin was detected in 1.88% of *H. pylori* positive cases. Old age (78-86) years was presented with duodenal ulcer 2.83%, they have 7 fold risk to get infection than those with *H. pylori* negative gastritis. Cag A cytotoxin detected in 2.83% of *H. pylori* positive cases.

As shown in Table 4, *H. pylori* detected in 34.91% in patients have no previous gastric medical history, 25.5% in patients previously suffered from gastritis, gastric ulcer 5.66%, duodenitis 6.60%, duodenal ulcer 2.8%. Significant difference between groups regarding *H. pylori* positivity (p value < 0.001), No significant correlation between previous medical history and recent *H. pylori* Infections (r = 0.093, p value = 0.34). No significant correlation reported between previous medical history and infection with *H. pylori* Cag A positive (r = 0.162, p value = 0.097). Patients come with major complains, weight loss represent 37.7%, mild heat burn 70.8%, moderate 8.5%, mild epigastric pain 44.3%, moderate 46.2%, anemia 21.7%, nausea 68.9%, mild bloating 25.5%, as shown in Table 4.

**Table 4:** Predictive clinical symptoms and major complains for *H. pylori* infection

Parameters			No. (%)	*H. pylori* test				X2 p value	Correlation with *H. pylori*		Correlation with *H. pylori* Cag A cytotoxin	
	
				Positive			Total *H. pylori* Negative		r	P value	r	P value

				Total *H. pylori* positive	Cag A cytotoxin positive	Cag A cytotoxin negative						
Previous Medical History		Negative history	55 (51.9%)	37 (34.91%)	19 (17.92%)	18 (16.98%)	18 (16.98%)	0.000	0.093	0.34	0.162	0.097

		Gastritis	31 (29.2%)	27 (25.5%)	17 (16.04%)	10 (9.43%)	4 (3.77%)					

		Gastric ulcer	7 (6.6%)	6 (5.66%)	6 (5.66%)	0 (0%)	1 (0.94%)					

		Duodenitis	8 (7.5%)	7 (6.60%)	3 (2.83%)	4 (3.77%)	1 (0.94%)					

		Duodenal ulcer	5 (4.7%)	3 (2.8%)	3 (2.83%)	0 (0%)	2 (1.89%)					

		Total	106 (100%)	80 (75.47%)	48 (45.28%)	32 (30.19%)	26 (24.53%)					

Major Complains	Weight loss	Positive	40 (37.7%)	37 (34.91%)	29 (27.36%)	11 (10.38%)	3 (2.83%)	0.012	0.308**	0.001	0.426*	0.000

		Negative	66 (62.3%)	43 (40.57%)	19 (17.92%)	24 (22.64%)	23 (21.7%)					

	Heartburn	Negative	22 (20.8%)	14 (13.21%)	11 (10.38%)	3 (2.83%)	8 (7.55%)	0.000	0.200*	0.040	0.104	0.289

		Mild	75 (70.8%)	57 (53.77%)	29 (27.36%)	28 (26.42%)	18 (16.98%)					

		Moderate	9 (8.5%)	9 (8.49%)	8 (7.55%)	1 (0.94%)	0 (0%)					

	Epigastric pain	Negative	10 (9.4%)	6 (5.66%)	5 (4.7%)	1 (0.94%)	4 (3.77%)	0.000	0.154	0.114	0.068	0.487

		Mild	47 (44.3%)	34 (32.08%)	18 (16.98%)	16 (15.09%)	13 (12.26%)					

		Moderate	49 (46.2%)	40 (37.74%)	25 (23.58%)	15 (14.15%)	9 (8.49%)					

	Iron deficiency anemia	Negative	83 (78.3%)	60 (56.60%)	34 (32.1%)	26 (24.53%)	23 (21.70%)	0.000	0.141	0.151	0.165	0.091

		Positive	23 (21.7%)	20 (18.86%)	14 (13.21%)	6 (5.66%)	3 (2.83%)					

	Nausea	Negative	33 (31.1%)	23 (21.70%)	16 (15.09%)	7 (6.60%)	10 (9.43%)	0.000	0.090	0.358	-0.043	0.660

		Positive	73 (68.9%)	57 (53.77%)	32 (30.19%)	25 (23.58%)	16 (15.09%)					

	Bloating	Negative	79 (74.5%)	63 (59.43%)	38 (35.85%)	25 (23.58%)	16 (15.09%)	0.000	0.093	0.341	-0.097	0.323

		Mild	27 (25.5%)	17 (16.04%)	10 (9.43%)	7 (6.60%)	10 (9.43%)					

* Correlation is significant at the 0.05 level (2-tailed);

** Correlation is significant at the 0.01 level (2-tailed).

A total 34.91% of patients with positive *H. pylori* suffered from weight loss while 40.57% of *H. pylori* positive cases never complain weight loss with significant difference (p < 0.05). Significant correlation reported between weight loss and *H. pylori* infection (r = 0.308, p value = 0.001). Significant correlation reported between weight loss and infection with *H. pylori* Cag A positive (r = 0.426, p value < 0.001), as shown in Table 4.

*H. pylori* positive patients presented with no history of heartburn represent 13.21%, 53.77% for mild and 8.49% for moderate heart burn with significant difference (p < 0.05) and correlation between heartburn and *H. pylori* infection (r = 0.200, p value = 0.040). No correlation reported between heartburn and infection with *H. pylori* Cag A positive (r = 0.104, p value = 0.289). *H. pylori* positive patients presented with no history of epigastric pain represent 5.66%, 32.08% for mild and 37.74% for moderate epigastric pain with significant difference (p < 0.05). No correlation between epigastric pain and *H. pylori* infection (r = 0.154, p value = 0.114). No correlation reported between epigastric pain and infection with *H. pylori* Cag A positive (r = 0.068, p value = 0.487). *H. pylori* positive patients presented with no history of anemia represent 56.60% and 18.86% of patients with positive *H. pylori* presented with anemia with significant difference (p < 0.05) but without correlation between anemia and *H. pylori* infection (r = 0.141, p value = 0.151). No correlation reported between anemia and infection with *H. pylori* Cag A positive (r = 0.165, p value = 0.091). *H. pylori* positive patients presented with no history of nausea represent 21.70% and 53.77% of patients with positive *H. pylori* presented with nausea with significant difference (p < 0.05), without correlation between nausea and *H. pylori* infection (r = 0.090, p value = 0.358). No correlation reported between nausea and infection with *H. pylori* Cag A positive (r = -0.043, p value = 0.660). *H. pylori* positive patients presented with no history of bloating represent 59.43% and 16.04% of patients with positive *H. pylori* presented with mild bloating with significant difference (p < 0.05), without correlation between bloating and *H. pylori* infection (r = 0.093, p value = 0.341).

No correlation reported between bloating and infection with *H. pylori* Cag A positive (r = -0.097, p value = 0.323).

As shown in Table 5, a total of 88.68% patients suffering from multiple pathological finding ranged from inflammation 83% to sever erosion 5.7%. lesions in antrum represent 34.9% the lesions located mainly in antrum in which 28.30% was *H. pylori* positive. Lesions in body and antrum were detected in 5.7% among them 4.72% *H. pylori* positive. Multiple lesions detected in 41.5%, among them 25.47% were *H. pylori* positive. Duodenal lesions detected in 15.1%, among them 14.15% were *H. pylori* positive. All prepyloric lesions were *H. pylori* positive 2.83%. Significant difference (p value < 0.001) between groups in *H. pylori* infections. No correlation between *H. pylori* infection and anatomical location (r = -0.37, p value = 0.708). No correlation reported between location of pathology and infection with *H. pylori* Cag A positive (r = -0.141, p value = 0.150).

**Table 5:** Predictive endoscopic and diagnostic findings for *H. pylori* infection

Parameters			No. (%)	*H. pylori* test				X2 p value	Correlation with *H. pylori*		Correlation with *H. pylori* Cag A cytotoxin	
	
				Positive			Total *H. pylori* Negative		r	P value	r	P value

				Total *H. pylori* positive	Cag A** cytotoxin** positive	Cag A** cytotoxin negative						
Endoscopic findings	Pathology Location	Antrum	37 (34.9%)	30 (28.30%)	19 (17.92%)	11 (10.38%)	7 (6.60%)	0.000	-0.37	0.708	-0.141	0.150

		Body & antrum	6 (5.7%)	5 (4.72%)	5 (4.7%)	0 (0%)	1 (0.94%)					

		Multiple	44 (41.5%)	27 (25.47%)	17 (16.04%)	10 (9.43%)	17 (16.04%)					

		Duodenum	16 (15.1%)	15 (14.15%)	5 (4.7%)	10 (9.43%)	1 (0.94%)					

		Prepyloric	3 (2.8%)	3 (2.83%)	2 (1.89%)	1 (0.94%)	0 (0%)					

	Mucosal finding	Normal	12 (11.3%)	11 (10.37%)	7 (6.60%)	4 (3.77%)	1 (0.94%)	0.000	-0.146	0.136	-0.070	0.477

		Sever erosion	6 (5.7%)	5 (4.72%)	2 (1.89%)	3 (2.83%)	1 (0.94%)					

		Inflammation	88 (83.0%)	64 (60.38%)	39 (36.79%)	25 (23.58%)	24 (22.64%)					

		Total	106 (100%)	80 (75.47%)	48 (45.28%)	32 (30.18%)	26 (24.53%)					

Diagnosis	Gastric Ulcer		18 (16.98%)	16 (15.09%)	17 (16.04%)	1 (0.94%)	2 (1.89%)	0.000	-.0372	0.000	-0.372**	0.000

	Gastritis		40 (37.74%)	27 (25.47%)	9 (8.5%)	31 (29.25%)	13 (12.26%)					

	Gastropathy		29 (27.36%)	19 (17.92%)	13 (12.26%)	16 (15.1%)	10 (9.43%)					

	Duodenal ulcer		13 (12.26%)	12 (12.26%)	7 (6.60%)	6 (5.7%)	1 (0.94%)					

	Duodenitis		6 (5.66%)	6 (5.66%)	2 (1.89%)	4 (3.77%)	0 (0%)					

* Correlation is significant at the 0.01 level (2-tailed);

** Correlation is significant at the 0.05 level (2-tailed).

A total of 60.38% of cases with mucosal inflammation was *H. pylori* positive v 4.72% with sever erosion and 10.37% normal mucosa. Significant difference (p value < 0.001) between groups in *H. pylori* infections, no correlation between *H. pylori* infection and anatomical location (r = -0.146, p value = 0.136). No correlation was reported between mucosal pathology and infection with *H. pylori* Cag A positive (r = -0.070, p value = 0.477), as shown in Table 5.

*H. pylori* gastritis detected in 25.47%, 8.5% was Cag A positive. *H. pylori* gastric ulcer detected in 15.09%, 16.04% was Cag A positive. *H. pylori* gastropathy detected in 17.92%, 12.26% was Cag A positive strains. *H. pylori* duodenal ulcer detected in 12.26% of patients, 6.60% was Cag A positive. Collectively there was significant difference between groups regarding *H. pylori* positivity (p value < 0.001). Inverse correlation between endoscopic diagnosis and recent *H. pylori* Infections (r = -.0372, p value < 0.001). Significant correlation reported between endoscopic diagnosis and infection with *H. pylori* Cag A positive (r = -0.372, p value < 0.001), as shown in Table 5.

**Figure 1:**
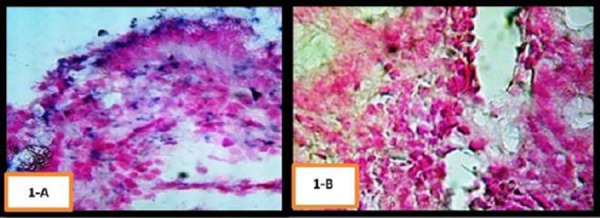
*A) In situ hybridization for human gastric ulcer tissue section. Staining by BCIP/NBT (bluish purple) counterstained with nuclear fast red. Bar size = 50 µm. B) CagA negative expressions in normal gastric tissue section*.

## Discussion

In the present study, The prevalence of *H. pylori* infection was 75.47% which was considered high compared with neighbours countries such as Saudi Arabia 49.8% [13]; Iran 34% [14] to 66.6% [15] while in India is 44.23% [16] to 93.3% [17]. The difference in *H. pylori* infection rate may be due to socio economic, demographic, cultural factors as well as healthier lifestyle differences among communities.

The mean age of patients was 44.70 years which come in accordance with others in Iraq [18]; Iran [15] and higher than Turkey 21.14 years [12]. *H. pylori* infection was detected in 22.64% among age group 33-41 years, suffered from gastropathy 10.37% and gastritis 7.55%. They have 3.77 fold risks to get *H. pylori* infection. CagA cytotoxin was detected in 16.04%. This come in agreement with a study in northern Iraq 22.08% [18]. In India the majority 50.7% of *H. pylori* positive cases at 30-39 years, followed by 40-49 years (45.9%) [16]. Similar conclusion was reported in Iran [19]. The second age group in *H. pylori* exposure was 24-32 years, in which 15.09% of them presented with gastritis 4.72% and gastropathy 3.77%. They have 2.2 fold to get *H. pylori* infection compared with *H. pylori* negative gastropathy or gastritis at the same age group. In northern Iraq the prevalence among this age group was 39.40% and 67.5% in other study [20]. In Turkey the prevalence among this age group was extremely lower (9.5%) [12] while in Iran the prevalence among health care workers < 30 years was 30.3% and among those of ≥ 30 years was 69.7% without significant difference in *H. pylori* exposure among age groups [14]. The third age group, which was more exposed to *H. pylori* was 69-77 years, suffered from gastric ulcer 6.60% and gastritis 4.72%. They have 6.20 fold to get *H. pylori* infection. This result considered extremely higher than in India in which only (20%) of patients was *H. pylori* positive at the age <70 years [16]. In current study, *H. pylori* Infection were detected in 7.55% among 42-50 years. Patients suffering from gastropathy were 3.77% and gastritis 2.83%. They have 3.4 fold risks to get *H. pylori* infection. In Indian study 42-50 years age group exposure to *H. pylori* infection represent 45.9 % [16]. The differences may be due to study design, nature of selected population, social and cultural factors.

*H. pylori* Infection was detected among 51-59 years in 6.60%, suffering from gastritis 5.66%, have 2.14 fold risks to get *H. pylori* infection. Old ages (78-86) years were presented with duodenal ulcer (2.83%) and they have 7 fold risk to get *H. pylori* infection. In India the prevalence of *H. pylori* among patients over 70 years old was 20% [16]. Significant difference was detected among age groups in *H. pylori* infection (p < 0.001). Positive correlation was reported between age groups and *H. pylori* infection (p value = 0.018). This come in line with others stated that *H. pylori* exposure increased and associated with age [20-22]. This result disagree with [14] reported that, no significant difference and relationship among *H. pylori* positive patients and age distribution [14], [23].

In current study, no correlation was reported between age and infection with Cag A producing *H. pylori*. This come in contrary with others found a strong correlation between age groups and infection with CagA positive *H. pylori* [24]. The differences between countries in *H. pylori* prevalence; in clinical disease caused by this pathogen related to the fact that prevalence of *H. pylori* varies widely by geographic area, age, race, and ethnicity and socioeconomical status as well as due to study design, nature of selected population, social and cultural factors [16].

In the present study, males have 1.38 fold risk of *H. pylori* acquisition compared with females with no significant differences between males and females (p > 0.05). There was no correlation between total *H. pylori* infection, CagA+ *H. pylori* infection and gender (p value = 0.492). This come in line with other study in the north of Iraq, they found that 47.3% of *H. pylori* positive patients were male versus 41.9% females with no significant difference in *H. pylori* acquisition [20]. Same results were reported in Saudi Arabia [22], China [25], Iran [14], and Turkey [12]. Although others consider female gender as a risk factor for *H. pylori* infection [19]. The reason for the possible gender difference in *H. pylori* prevalence is unclear but may be related to the frequency of eating in restaurants and smoking [22].

In this study, people’s residence in rural areas has (0.63) fold risk of *H. pylori* acquisition compared with those from urban without significant difference (p > 0.05). No correlation was reported between total *H. pylori* infection, *H. pylori* Cag A+ and residency (p value = 0.317) (p value = 0. 501). This come in line with other study in Iraq [20] and Iran [26]. In Saudi Arabia residency in rural areas considered as a risk for *H. pylori* infection [22]. The high prevalence of *H. pylori* infection in rural areas may reflect the fact that low socioeconomical status and absent of sanitary measures beside cultural variation in local community may have an important role in this pattern. In the present study, there was significant differences between smokers infected with *H. pylori* and those with negative tests for *H. pylori* (p < 0.05), although there was no correlation between total *H. pylori* infection, *H. pylori* CagA + and smoking (p value = 0.764). Nonsmokers with positive *H. pylori* tests represent 47.17% of total patients which is higher than in Saudi Arabia [22] and less than that reported in India 92.85% [17]. In the present study smokers whether (mild, moderate or heavy) with positive *H. pylori* tests constitute 28.30% and 16.04% Cag A+ which is less than India 93.75% [17] and Saudi Arabia 57.9%. In this study, mild smokers have 18 fold risk to get *H. pylori* infection and 20.75% were positive for *H. pylori*, moderate smokers have 1.4 fold risk for *H. pylori* infection and 6.60% were *H. pylori* positive while heavy smokers have one fold risk and have 0.94% positive *H. pylori*. This come agreement with local study [18]. The possible mechanism for increase infection among smokers, in general may attributed to the destructive effect of smoking on the immunity of the gastric mucosa and lining layers, hence increasing their susceptibility to infection by *H. pylori*. Communal Shisha smoking might carry the risk of passing the infection from a diseased person to an uninfected one, in the form of oral infection [22]

Peoples whose in contact with animals have 1.52 fold risk of *H. pylori* acquisition compared with those who do not in direct contact with animals with significant difference (p < 0.05) although no correlation between *H. pylori* infection and animals contact (p value = 0.431). This result confirmed by Italian study referred to detection of *H. pylori* phosphoglucosamine mutase gene in 34.7% of raw goat milk samples [27] and in Japan ureA gene of *H. pylori* was detected in 72.2% raw milk samples and in 11/20 (55%) commercial pasteurized milk samples [28]. It is clear from present results that goat’s or cow’s milk considered as a major factor for *H. pylori* transmission. In addition, since the host factor is important in colonization of *H. pylori* in the human, the infection may be established in a subset of individuals who drink goat’s or cow’s milk.

Illiterates and patients with primary education have 5.36 & 3 fold risk for acquisition of *H. pylori* infection. While those at secondary and university education level have, 0.42 and 1.24 fold risk, respectively without significant differences (p > 0.05). Inverse correlation was reported between *H. pylori* infection and education levels (p value = 0.020). This come in agreement with others, stated that educated patients had a lower frequency of *H. pylori* infection compared with those who were illiterates [29]. Education level not necessarily reflects higher socioeconomic status for patients and this explanation come in contrary with others, indicated that individuals of higher socioeconomic status are often less likely to be infected with *H. pylori* due to hygienic standards of their life styles [30].

Patients under PPI therapy have 1.02 risk fold of *H. pylori* infection compared with PPI negative with significant difference (p < 0.05). No correlation between PPI intake and *H. pylori* infection (p value = 0.968). This finding may be due to the mechanism of PPIs which are selectively inhibits the gastric H^+^-K^+^ATPase and hence gastric acid secretion. All PPIs irreversibly inhibits the gastric H^+^-K^+^ATPase by binding to alpha subunit of the proton pump. Both basal and stimulated secretion of gastric acid is inhibited, independent of the nature of parietal cell stimulation. Gastric acid at pH < 4 has a powerful bactericidal effect, capable of killing exogenous acid sensitive bacteria introduced in to the stomach usually within 15 min [31]. Any increase in the gastric pH above 4 due to PPI causes a state of hypochlorhydria and potentially increases the susceptibility to various microbes, allowing at least 50% of ingested bacteria to survive within the gastric trap [31].

Patients under NSAID therapy have 3.48 fold risk of *H. pylori* infection compared with NSAID negative cases with significant difference (p < 0.05). No correlation between NSAID intake and *H. pylori* infection (p value = 0.095). This comes in line with [17], stated that no correlation between *H. pylori* status and NSAID intake. Others reported a negative interaction between *H. pylori* and NSAID intake on duodenal ulcers suggesting that *H. pylori* reduces the development of these ulcers in NSAID users [32, 33]. Accumulated evidence shows that both aspirin and *H. pylori* upregulate the expression of cyclooxygenase (COX)-2 at both mRNA and protein levels at the ulcer margin. It was, therefore, proposed that *H. pylori* may in fact, antagonize, aspirin-induced delay of ulcer healing due to suppression of acid secretion by the enhancement in PGE2 possibly derived from COX-2 expression and activity and to the overexpression of growth factors such as TGFα and VEGF [33].

Nonalcoholic Patients have 0.75 risk fold of *H. pylori* infection compared with alcoholics with significant difference (p < 0.05), without significant correlation between alcohol intake and *H. pylori* infection (p value = 0.056). This comes in line with [17], stated no correlation between *H. pylori* status and alcohol intake. In contrary others investigate the association between *H. pylori* and alcohol intake with conflicting results [34]. While others considered alcohol consumption a significant independent predictor for *H. pylori* infection [22]. The mechanisms that would promote an association with alcohol intake were unclear.

Patients drinking spigot water have 0.45 fold to get *H. pylori* infection compared with filtrated water with significant difference (p < 0.05). No correlation between drinking of spigot water and *H. pylori* infection (p value = 0.236). This comes in line with others stated that significant association between the presence of *H. pylori* and clinical infection in individuals drinking the tap water [35]. Evidence for water as a vehicle for transmission has been provided by maintenance of *H. pylori* viability in water [36], amplification of *H. pylori*-specific nucleic acid sequences in water [37] and detection of actively respiring *H. pylori* in surface and groundwater [38].

In current study, no correlation was found between previous medical history, recent *H. pylori* Infections (p value = 0.34), and Cag A positive *H. pylori* (p value = 0.097) which comes in line with [39].

Significant difference (p < 0.05) and correlation between weight loss and *H. pylori* infection (p value = 0.001) was reported. This comes in line with local study [23] and in contrary with others in Iran [39]. In present study significant correlation was reported between weight loss and infection with Cag A positive *H. pylori* (p value < 0.001), no current or previous study reports this fact. Significant difference (p<0.05) and correlation between heartburn and *H. pylori* infection (p value = 0.040) was reported, this comes in agreement with other studies in Iraq [23], [29]. Others, reported that heartburn was absent after *H. pylori* eradication [40] in contrary, others found no such difference or correlation [39]. No correlation was reported between heartburn and infection with Cag A positive *H. pylori* (p value = 0.289) which comes in line with [41]. Current study was reported significant difference (p < 0.05) without correlation between epigastric pain and *H. pylori* infection (p value = 0.114) which comes in line with other local study [23]. In Iranian study no such difference was detected [39]. No correlation was reported between epigastric pain and infection with Cag A positive *H. pylori* (p value = 0.487) which comes in accordance with others [41] and in contrary with others [29].

Current study reported significant difference (p < 0.05) without correlation between anemia, *H. pylori* infection (p value = 0.151), and Cag A positive *H. pylori* (p value = 0.091). This comes in line with [42] and in contrary with [43, 44] who found significant correlation between iron deficiency anemia and *H. pylori*. As significant difference among *H. pylori* infected and non-infected group present, although no correlation was reported with anemia but this reflect the fact that the majority of patients suffering from weight loss which correlated with the anemic status of patients. Significant difference (p < 0.05) without correlation between nausea and *H. pylori* infection was reported (p value = 0.358) which comes in line with [23]. No correlation was reported between nausea and infection with Cag A positive *H. pylori* (p value = 0.660) which comes in contrary with [41] [29]. Significant difference (p < 0.05) without correlation between bloating and *H. pylori* infection (p value = 0.341) was reported which comes in line with other [23]. No correlation was reported between bloating and infection with Cag A positive *H. pylori* (p value = 0.323) which come in contrary with others [29].

In current study, significant difference (p value < 0.001) between groups in *H. pylori* infections without correlation between *H. pylori* infection and anatomical location of lesions whether in antrum, body and antrum or multiple sites (p value = 0.708), which comes in line with [23]. No correlation was reported between location of *H. pylori* associated pathology and infection with Cag A positive *H. pylori* (p value = 0.150) which comes in contrary with others found significant correlation between CagA and gastric and duodenal ulcers [45]. No correlation was reported between mucosal pathology and infection with Cag A positive *H. pylori* (p value = 0.477) which comes in contrary with [45], found significant correlation between CagA, gastric and duodenal ulcers. Other found that the correlation between *H. Pylori*, mucosal pathology, anatomical location in gut, and the infection with *H. pylori* can be estimated via clinical features of infected mucosal lining [46]. Inverse correlation was reported between endoscopic diagnosis and recent *H. pylori* Infections (p value < 0.001). A correlation was reported between endoscopic diagnosis and infection with Cag A positive *H. pylori* (p value < 0.001) which comes in line with [45, 47].

In conclusion, several life style factors, education, animal contact, using of PPI, and NSAIDs increase the risk of *H. pylori* infection. Weight loss and heartburn considered as a cardinal signs for *H. pylori* infection. Endoscopic diagnosis and clinicopa-thological parameters not strictly associated with CagA positivity.
